# Internal Thoracic Impedance - A Useful Method for Expedient Detection and Convenient Monitoring of Pleural Effusion

**DOI:** 10.1371/journal.pone.0122576

**Published:** 2015-04-28

**Authors:** Gideon Charach, Olga Rubalsky, Lior Charach, Alexander Rabinovich, Ori Argov, Ori Rogowski, Jacob George

**Affiliations:** 1 Department of Internal Medicine “C”, Tel Aviv Sourasky Medical Center, affiliated to the Sackler Faculty of Medicine, Tel Aviv University, Tel Aviv, Israel; 2 Cardiology Department Kaplan Medical Center, affiliated to Hebrew University Medical Center, Jerusalem, Israel; Kurume University School of Medicine, JAPAN

## Abstract

Measurement of internal thoracic impedance (ITI) is sensitive and accurate in detecting acute pulmonary edema even at its preclinical stage. We evaluated the suitability of the highly sensitive and noninvasive RS-207 monitor for detecting pleural effusion and for demonstrating increased ITI during its resolution. This prospective controlled study was performed in a single department of internal medicine of a university-affiliated hospital between 2012-2013. One-hundred patients aged 25–96 years were included, of whom 50 had bilateral or right pleural effusion of any etiology (study group) and 50 had no pleural effusion (controls). ITI, the main component of which is lung impedance, was continuously measured by the RS-207 monitor. The predictive value of ITI monitoring was determined by 8 measurements taken every 8 hours. Pleural effusion was diagnosed according to well-accepted clinical and roentgenological criteria. During treatment, the ITI of the study group increased from 32.9±4.2 ohm to 42.8±3.8 ohm (*p*<0.0001) compared to non-significant changes in the control group (59.6±6.6 ohm, *p* = 0.24). Prominent changes were observed in the respiratory rate of the study group: there was a decrease from 31.2±4.0 to 19.5±2.4 ohm (35.2%) compared to no change for the controls, and a mean increase from 83.6±5.3%-92.5±1.6% (13.2%) in O2 saturation compared to 94.2±1.7% for the controls. Determination of ITI for the detection and monitoring of treatment of patients with pleural effusion enables earlier diagnosis and more effective therapy, and can prevent hospitalization and serious complications, such as respiratory distress, and the need for mechanical ventilation.

**Trial Registration:**

The study is registered at ClinicalTrials.gov NCT01601444

## Introduction

Previous studies have shown the suitability of the RS-207 monitor (R. S. Medical Monitoring, Jerusalem, Israel) for measuring internal thoracic impedance (ITI) and enabling early diagnosis of pulmonary edema [1–15]. No studies have dealt with early diagnosis of pleural effusion by this simple, noninvasive and inexpensive method. Radiographic examinations are widely used for detecting pleural effusion, but are not suitable for prolonged monitoring of patients at high risk of developing it. Moreover, the currently available methods for the early detection and monitoring of pulmonary edema were not studied for pleural effusion and are not sufficiently reliable, and they themselves may lead to complications [1–10]. Yu et al. [9] reported successful prediction of cardiogenic pulmonary edema (CPE) by a surgically implanted impedance plethysmograph integrated into a pacemaker. However, since these methods are invasive and not suitable for widespread use [9], they are seldom employed for the detection of pulmonary effusion [2–7]. The changes in the blood and extravascular fluid content in the lungs in humans and animals can be easily monitored by a noninvasive procedure based on impedance plethysmography of the right lung [8–15]. Total transthoracic impedance (TTI) consists of ITI and skin contact impedance. The TTI of the right lung may vary from 920 to 1230 ohm among different individuals [13], however, changes of TTI in pulmonary edema were reported to vary between 2–16 ohm [7–9,15–18], which is approximately 1% of TTI and 1.5% of skin contact impedance. Monitors used in previous works were not sensitive enough to detect the relatively small changes in pulmonary impedance [2–7].

One impedance monitor, the Edema Guard Monitor (EGM) model RS-207 (R. S. Medical Monitoring, Jerusalem, Israel [12]), unlike the other currently available impedance monitors, measures only the ITI of the right lung, which roughly equals lung impedance, by automatically calculating skin electrode impedance and subtracting it from the TTI [8,15].

Its results showed much higher sensitivity compared with the 1.5% changes found when using TTI methods. Measurement of ITI of the left lung is less accurate because the heart and great arteries make up a considerable body of fluids that tends to decrease the ITI value. This is especially prominent in patients with cardiomyopathy where the heart is enlarged mostly because of left ventricular dilatation.

This monitor was successful for the early detection of pulmonary edema [13–21], but there have been no reports describing its application for detecting pleural fluid. The objective of our study was to evaluate the suitability of the RS-207 in the detection and monitoring of pleural effusion during both the preclinical and clinical stages.

## Materials and Methods

**Fig 1:**
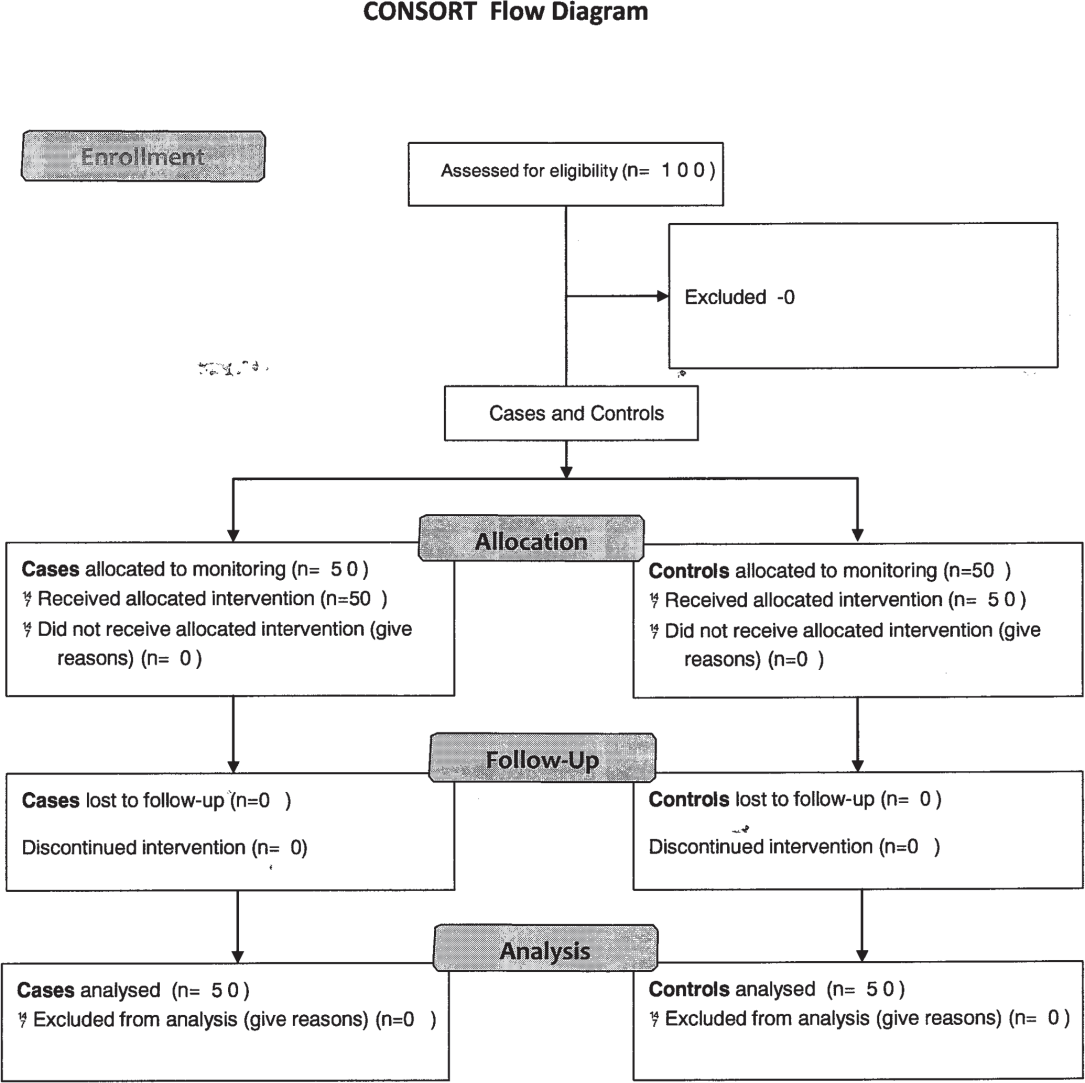
Consort Flow Diagram.

We designed a prospective controlled study between July 2012—August 2013 on 50 consecutive patients diagnosed as having pleural effusion and admitted to the Department of Internal Medicine “C” of the Tel Aviv Medical Center using an individual matching approach. Fifty carefully age-matched patients with various diagnoses without evidence of pleural effusion on their chest X-rays were selected from the same department and served as the control group. Inclusion criteria for the study group were congestive heart failure, valvular heart disease, renal failure, infectious disease complicated by pleural effusion, and malignant diseases that were complicated by bilateral or right lung pleural effusion The exclusion criteria were wearing a pacemaker, thoracic deformation, pregnancy, coma and respiratory failure due to diseases, i. e., pulmonary edema and embolism (Fig 1). The protocol of the investigation corresponded to the principles outlined in the Declaration of Helsinki, and the study protocol was approved by the local Ethics Committee of the Tel Aviv Medical Center and the Israeli Ministry of Health, number 0504-11-TLV. The protocol for this trial and supporting CONSORT checklist are available as supporting information; see S1 CONSORT Checklist and S1 Protocol. The study is registered at ClinicalTrials.gov with the registration number NCT01601444. All the participants gave written informed consent before being enrolled in the study. The authors confirm that all ongoing and related trials for this intervention are registered.

The diagnosis of pleural effusion was based on the following symptoms and signs: progressive dyspnea at rest (20 respirations), tachycardia (>90 beats /min), diaphoresis, cyanosis, dullness on percussion, crepitation rales, roentgenographic evidence of pleural effusion and arterial hypoxemia (<92%) [15]. ITI measurements were taken continuously. Clinical examination, as well measurement of additional parameters that correlate with clinical severity and improvement after treatment of pleural effusion, including oxygen saturation (O_2_%), respiratory rate (RR), pulse rate, systolic (sBP) and diastolic blood pressure (dBP) was done every 8 hours until monitoring was terminated when successful conservative treatment was achieved or a pleural puncture was performed. A follow-up chest x-ray was performed thereafter.

Patients with pleural effusion were treated either conservatively (mainly based on the diuretics furosemide and Aldospirone) or by right lung pleural paracentesis. Monitoring of pleural effusion was carried out by placing three electrodes in the front and three electrodes in the back of the right thorax [8]. The result is the value of internal thoracic electrical resistance (impedance), the ITI. The method by which ITI is estimated is described in detail elsewhere [8,15].

## Statistical analysis

Calculating sample size for a confidence level of 95 and a confidence interval of 15 that represent target effect size of 35% found a group size of 45 to have the power to detect a significant change at a 2-tailed level of 0.05. Comparison of categorical parameters between the groups was done by the Pearson chi-square test or Fisher’s exact. The two-sample paired t-test was used to compare the two groups with respect to clinical parameters measured at baseline. T-tests were used to evaluate the difference between all clinical parameters measured in the first and in the last visit. Differences were analyzed for each group (first visit vs. last visit as well as between groups in each of these visits. Pearson correlation coefficients were calculated between all parameters per each time point to examine the relationships between the two measurements. A *p* value of <0.05 was considered significant. Statistical analysis was performed by SAS for windows version 9.2.

## Results

The study cohort included 50 consecutive patients with pleural effusion (35 females and 15 males, mean age 73.8±13.8 years, range 25–96 years) and 50 patients without pleural effusion (35 females and 15 males, mean age 72.3±15.7 years, range 33–92 years) who served as controls. Their mean follow-up was 3.0 days (range 1.5–11.5 days). ITI values in the study and control groups, the primary comparison, are presented in Table 1, which also shows auxiliary measurements (O_2_%, RR, pulse rate, sBP, dBP) that helped to understand ITI variations in various stages of the clinical setting.

**Table 1:** Baseline ITI, oxygen%, RR, pulse, sBP, dBP, and BMI measurements of the study participants.

Group	ITI (ohms)	Oxygen Saturation (%)	Respiration (rate/min)	Pulse (rate/min)	sBP (mmHg)	dBP (mmHg)	BMI
**Study**	32.9±4.2	83.6±5.3	31.2±4.0	96.7±7.4	159.5±14.6	85.1±8.0	26.5±5.3
**Controls**	59.9±6.6	94.2±1.74	14.8±3.9	71.6±11.6	139.8±9.5	76.4±16.5	30.6±3.9
***p* value**	<0.0001	<0.0001	<0.0001	<0.0001	<0.001	<0.001	<0.0001

ITI: internal thoracic impedance; sBP: systolic blood pressure; dBP: diastolic blood pressure; BMI: body mass index.

The etiologies of pleural effusion in the study group were heart failure due to ischemic or valvular cardiomyopathy (34%), malignant disease (46%), parapneumonic fluid or empyema (13%), uremic pleural effusion (5%), and pericarditis (2%). The mean left ventricular ejection fraction of patients with heart failure was 47.7%, and the mean NYHA class was 2.8. Thirty-seven percent of the patients had a confirmed diagnosis of non-insulin-dependent diabetes mellitus, 58% had hypertension and 29% were current or past smokers. Thirty-six of the 50 study patients underwent pleural puncture. The 50 control patients were diagnosed as having ischemic heart disease (31%), infectious diseases, i.e., urinary tract infections, pneumonia/bronchitis, or cellulitis (35%), malignant disease (23%), and anemia (11%).

Body mass index (BMI) differed significantly between the study and control groups (26.5±5.3 versus 30.6±3.9, respectively, *p*<0.0001), and this difference persisted during the hospitalization period and throughout treatment.

**Fig 2:**
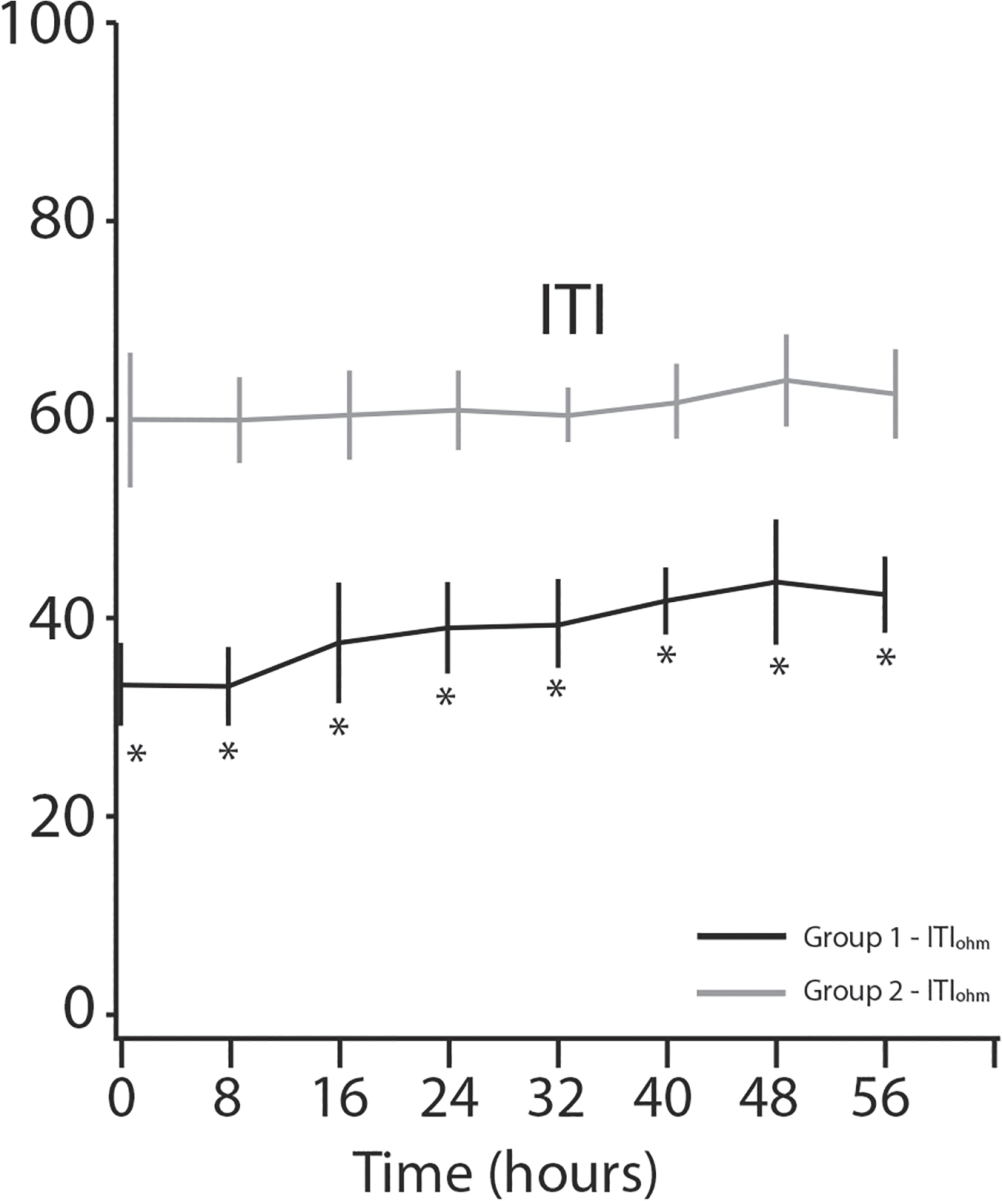
Internal thoracic impedance (ITI) changes over time. *P-value < 0.0001. **P-value < 0.01. ***P-value = non-significant.

**Fig 3:**
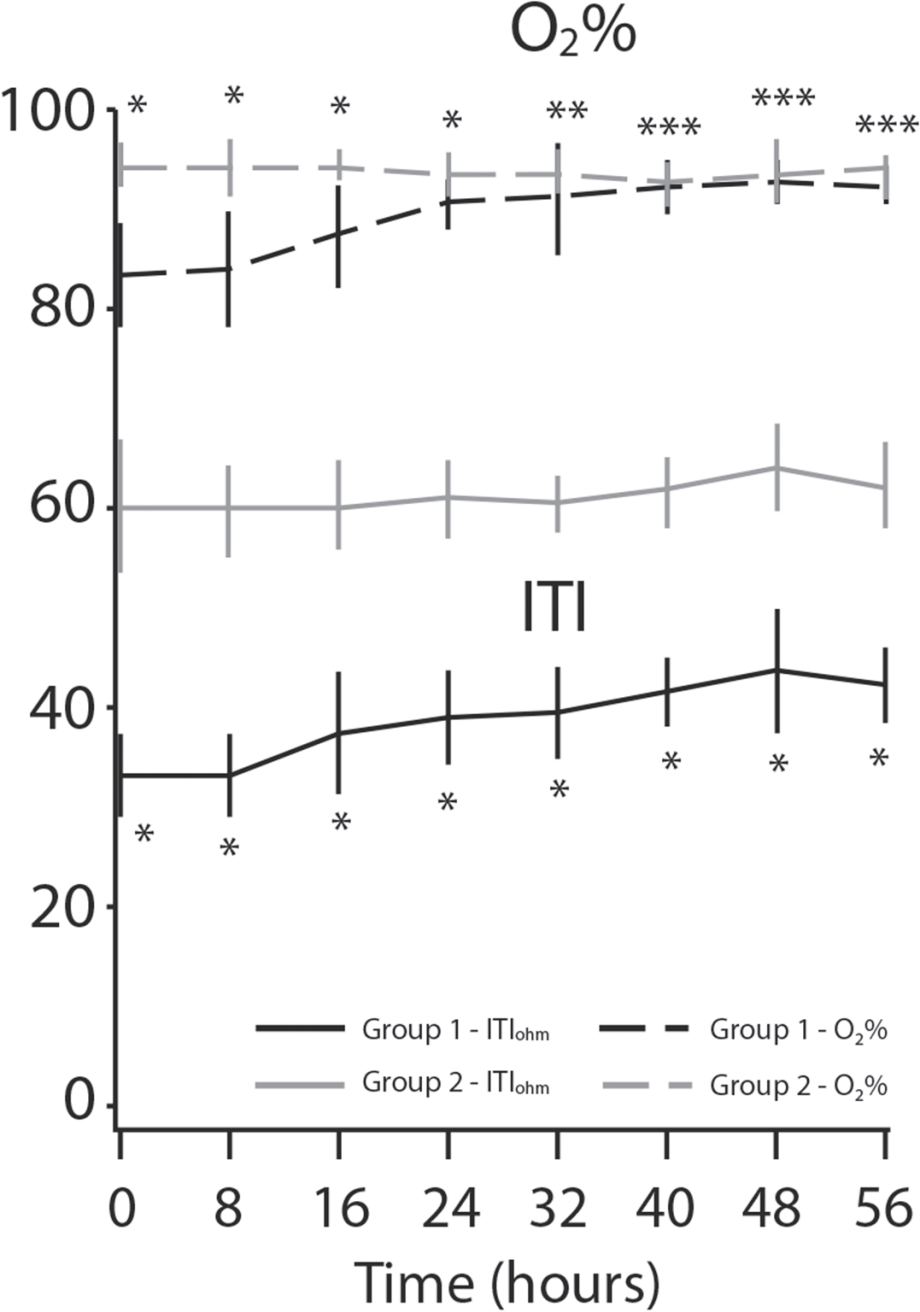
Oxygen saturation and internal thoracic impedance (ITI) changes over time. *P-value < 0.0001. **P-value < 0.01. ***P-value = non-significant.

ITI was measured continuously in all 100 participants. Values at time zero and at 8-hour intervals entered analysis. We used 8 measurements with a total monitoring of 56 hours to explore the behavior of ITI (note: changes in ITI are slow, even after pleural puncture). Fig **2** shows a significant increase in ITI following treatment of patients in the study group. At the beginning of monitoring mean ITI in the study group was 32.9±4.2 ohm compared to 59.6±6.6 ohm in the control group (*p*<0. 01). During treatment, mean ITI increased by 9.9 ohm (31.5%, *p*<0. 0001) in the study group, while there were no changes (*p* = 0.24) in the control group during the same monitoring period. Parameters that correlated with clinical severity and improvement after treatment of pleural effusion were measured, and they included oxygen saturation (O_2_%), RR, pulse rate, sBP and dBP. As expected, a prominent increase was seen in the O_2_% level only for the study group: the baseline mean O_2_% saturation of 83.6±5.6% rose to 92.5±1.6% at the end of the monitoring compared to 94.2±1.7and 93.6±0.1%, respectively, for the controls. Fig 3 shows O_2_% saturation and ITI values during 56 hours of monitoring.

**Fig 4:**
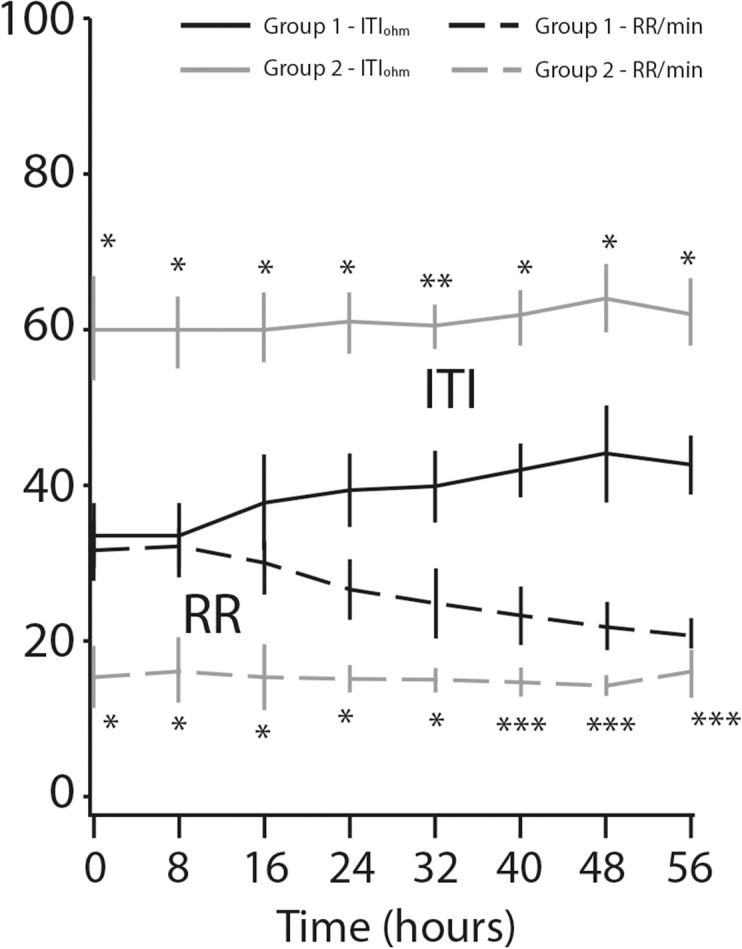
Respiratory rate (RR) and internal thoracic impedance (ITI) changes over time*P-value < 0.0001. **P-value < 0.01. ***P-value = non-significant.

**Fig 5:**
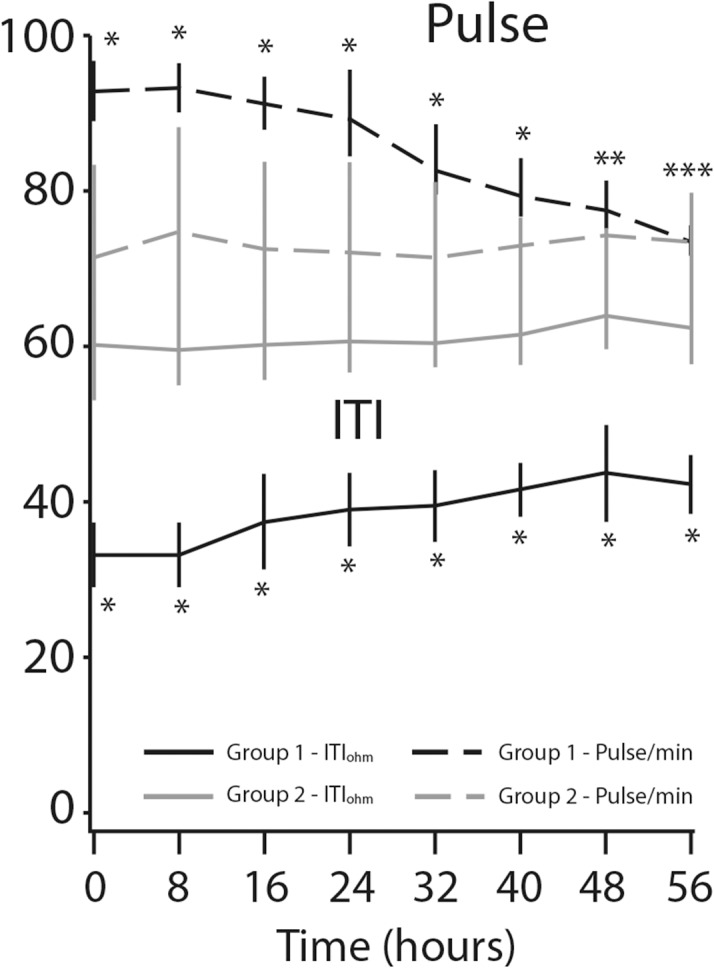
Pulse rate and internal thoracic impedance (ITI) changes over time. *P-value < 0.0001. **P-value < 0.01. ***P-value = non-significant.

**Fig 6:**
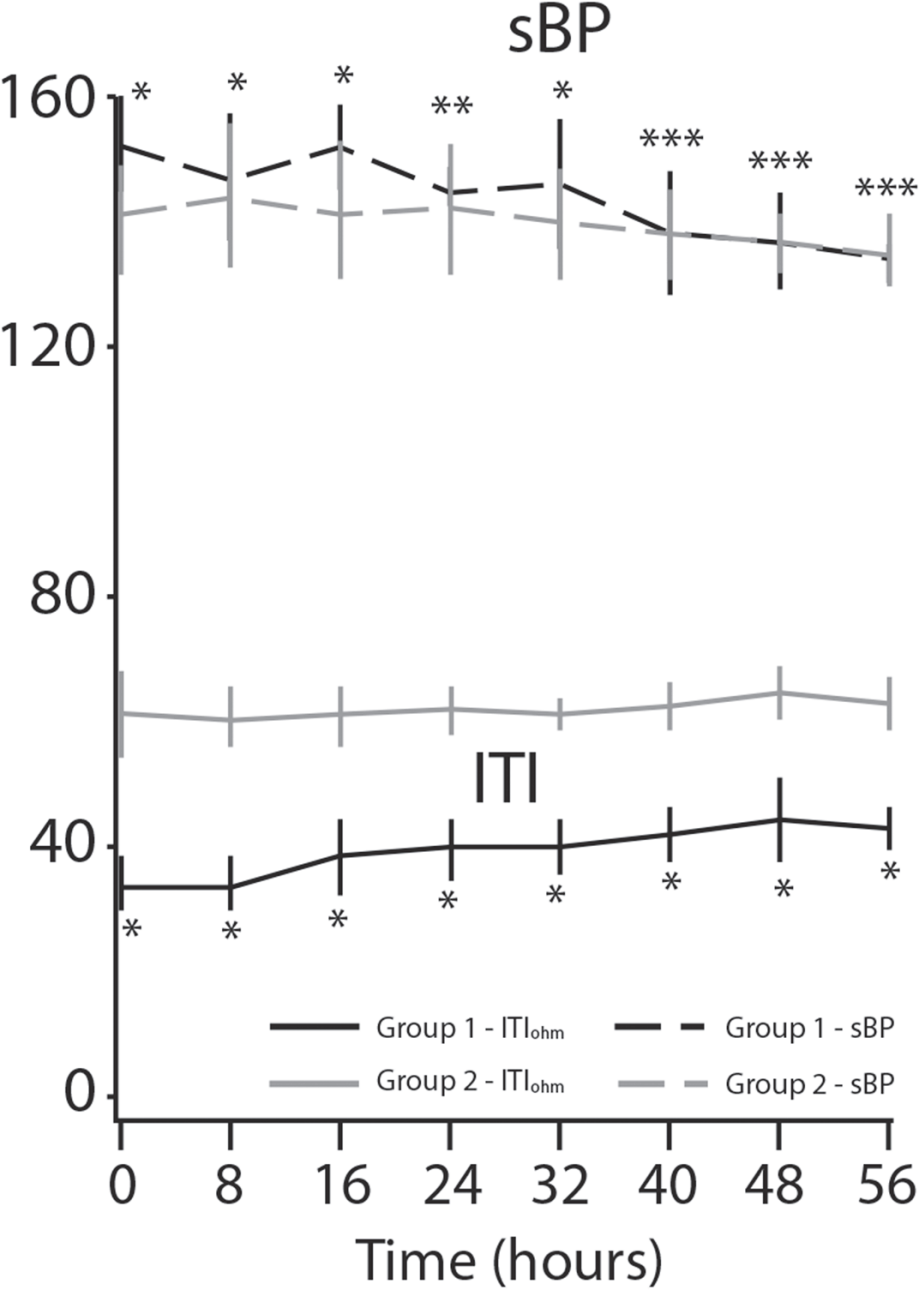
Systolic blood pressure (sBP) and internal thoracic impedance (ITI) changes over time. *P-value < 0.0001. **P-value < 0.01. ***P-value = non-significant.

**Fig 7:**
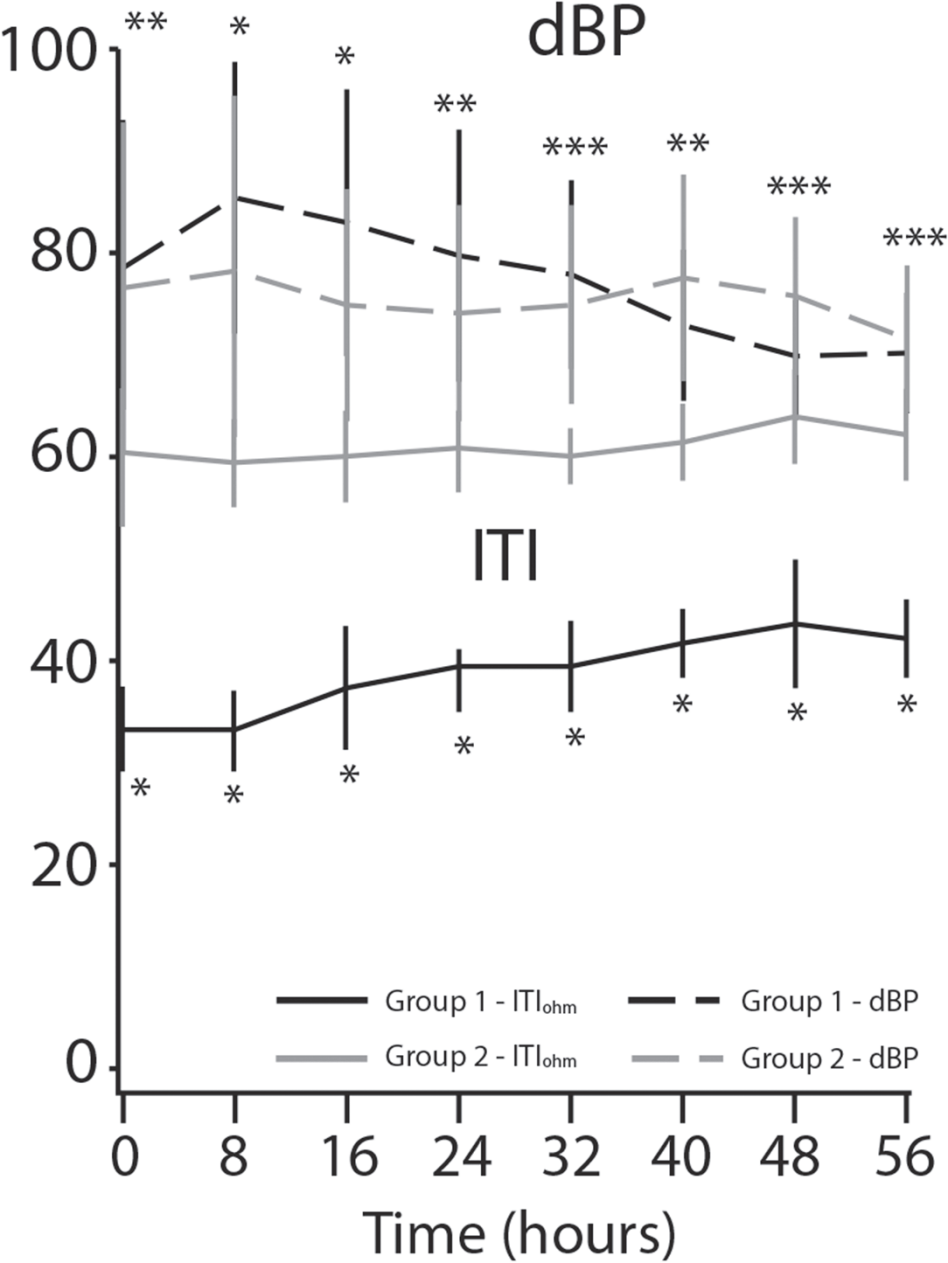
Diastolic blood pressure (dBP) and internal thoracic impedance (ITI) changes over time. *P-value < 0.0001. **P-value < 0.01. ***P-value = non-significant.

We found remarkable variation in respiratory rate between the two groups (Fig 4). The mean respiratory rate decreased from 31.2±4.0 per minute to 19.5±2.4 per minute (*p*<0.0001) in the study patients, while it remained unchanged (14.8±3.9 per minute) for the controls (*p* = 0.28). Pulse rate decrease was observed only in the study group of patients: mean baseline pulse rate of 96.7±7.4 per minute went down to 73.5±5.0 per minute after treatment, representing a decrease of 31% (*p*<0.0001) the respective values for the controls were 71.6±9.6 to 73.1 ±6.3 per minute (*p* = 0.31) (Fig 5). The same trend was found in sBP: a mean of 159.5±14.6 mmHg at baseline fell to 133.9±4.7 mmHg at the eighth measurement, representing a decrease of 18.6% (*p*<0.0001) for the study group. However, decrease in sBP was also seen in the control group (*p*<0.01) (Fig 6). Fig **7** displays the significant changes in dBP in both groups, which were more remarkable in the study group. Table 2 summarizes the differences between the values of the clinical parameters at baseline (time 0 hours) and at the end of treatment and monitoring (time 56 hours).

**Table 2:** Differences between the values of clinical parameters at baseline and at the end of monitoring.

	ITI (ohm)	ITI (ohm)	Oxygen (%)	Oxygen (%)	Respiratory (rate/min)	Respiratory (rate/min)	Pulse (rate/min)	Pulse (rate/min)	sBP (mmHg)	sBP (mmHg)	dBP (mmHg)	dBP (mmHg)
	Time 0 hours	Time 56 hours	Time 0 hours	Time 56 hours	Time 0 hours	Time 56 hours	Time 0 hours	Time 56 hours	Time 0 hours	Time 56 hours	Time 0 hours	Time 56 hours
**Group 1 (n = 50)**	32.9±4.2	42.8±3.8*	83.6±5.3	92.5±1.6*	31.2±4.0	19.5±2.4*	96.7+7.4	73.5±50*	159.5±14.6	133.9±4.7*	85.1±8.0	69.8±6.5*
**Group 2 (n = 50)**	59.6.±6.6	60.4±4.5***	94.2±1.74	93.6±0.1***	14.8±3.9	14.3±3.1***	71.6±9.6	73.1±6.3**	139.8±9.5**	135.2±5.5	76.4±9.5	71.7±7.1**

ITI: internal thoracic impedance; sBP: systolic blood pressure; dBP: diastolic blood pressure;

^*^ *p*<0.0001;

^**^ *p*<0.01;

^***^ *P*-value >0.05.

Special attention was directed to a group of 36 patients who underwent right-sided pleural paracentesis with a mean drained volume of 960 mL. As expected, these patients showed faster elevation in ITI (mean of 5.6 ohm) at the end of the first hour after the procedure, compared to a mean of 2.8 ohm for the entire group. In the group of patients with paracentesis, the mean overall ITI elevation at the end of 56 hours was 11.6 ohm, which was 33.7% higher (*p*<0. 01) than the ITI rise of 9.9% in the entire group (31.5%, *p*<0.01). Levels of O_2_%, RR, pulse rate, sBP and dBP were also quicker to improve.

The Pearson correlation coefficients of the ITI level at baseline with various clinical parameters were as follows: O_2_% = 0. 83 (*p*<0.01), RR = 0.87 (*p*<0.01), pulse = 0.77(*p*<0.01), sBP = 0.55 (*p*<0. 01), and dBP = 0.34 (*p*<0.01). Correlation remained high during 32 hours of monitoring and treatment and declined thereafter. The rise in ITI was related to the fluid volume drained by pleurocentesis (R^2^ = 0.21, *p* = 0.048).

Table 3 shows the distribution of sensitivity and specificity of the baseline ITI values measured in the study group. While the highest ITI was, obviously, associated with minimal sensitivity and maximal specificity and vice versa for the lowest ITI, both sensitivity and specificity values for ITIs in the range of 50–38 ohms were remarkably high (0.84–1). Indeed, most participants (72%) in this study had ITI values in the range of 50–38 ohms.

**Table 3:** Sensitivity and specificity of baseline ITI values.

ITI (ohm)	Sensitivity	Specificity
68	0.02	1.0
67	0.04	1.0
66	0.20	1.0
65	0.26	1.0
63	0.28	1.0
62	0.36	1.0
61	0.56	1.0
60	0.62	1.0
59	0.66	1.0
58	0.80	1.0
50	0.84	1.0
49	0.90	1.0
48	0.94	1.0
46	0.96	1.0
44	0.98	1.0
42	1.0	0.94
38	1.0	0.84
36	1.0	0.82
35	1.0	0.62
34	1.0	0.46
33	1.0	0.42
31	1.0	0.30
30	1.0	0.20
28	1.0	0.04
24	1.0	0.00

## Discussion

Pleural effusion is the result of progressive accumulation of fluid in the pleural space, leading to a decrease of pulmonary bio impedance. The significance of the current study, unlike earlier ones that dealt with the diagnosis of pleural effusion [8–21], is that its results demonstrate that continuous ITI monitoring of patients with pulmonary effusion is a highly precise method for diagnosis and for predicting respiratory deterioration, with the intent of implementing appropriate preventive therapeutic management in the future.

We studied the kinetics of ITI in patients admitted to the hospital for symptomatic pleural effusion. A decrease in ITI of approximately 12–21% was previously shown to reflect the maximal capacity of lung interstitium to accumulate fluid and to predict impending pulmonary edema [15–20]. Our current results revealed that the ITI levels in the presence of pleural effusion were significantly lower by approximately 40% (26 ohm) compared to those of control patients without pleural effusion. The decrease in the ITI threshold in CPE that we had reported earlier [15] yielded a high sensitivity (97.5%) and specificity (98%) of the EGM RS-207. The threshold was even higher when pleural effusion was monitored. The most prominent values were reached at ITI 44 ohm where sensitivity and specificity were the highest, i.e., 98% and 100%, respectively. ITI values in the wide range of 50-38ohm yielded reliable sensitivity and specificity that included all participants with ITI falling within 2 standard deviation of the mean (72% of the patients).

ITI of symptomatic patients was exceedingly low: specifically, it was a mean of 32.9 ohm in comparison to 59.5 ohm for the controls (representing a difference ratio of 31.3%). We had previously measured a 22-ohm decrease in ITI in cases of overt severe pulmonary edema [15]. We now measured a 26-ohm decrease in the ITI in cases of pleural effusion. These results indicate that ab ITI measurement using RS-207 is a reliable method that may be widely applicable in clinical setting of pleural effusion. Support for this contention came from the dynamic changes, i.e. improvement in heart rate, RR, O2% saturation as well as amelioration of respiratory distress that paralleled the disappearance of fluid from the right lung pleural space and elevation of ITI.

While ITI is not helpful in differentiating pleural effusion from pulmonary edema, it can be used to distinguish pleural effusion from other causes of dyspnea (e.g., chronic obstructive pulmonary disease, asthma, pulmonary emboli). The definitive diagnosis is made, however, by X-ray. In any event, ITI measurement is aimed primarily at monitoring patients with recurrent pleural effusion in order to administer early treatment.

Our current study and control groups were similar in age, however there were significant differences in their BMIs. An obese patient could be expected to have a higher TTI but not a higher ITI. A special mathematic algorithm using the equation, with 3 electrodes from each side of the chest, subtracts chest wall impedance (including subcutaneous adipose tissue) from the TTI [8]. The current findings failed to show any impact of BMI on ITI. The patients in the current study group received the treatment by diuretics or pleural puncture according to clinical signs and symptoms, and roentgenological findings. The ITI measurements showed clinically positive changes in impedance (i.e., an elevation of 31.3%) following the treatment. Typical direct or inverse changes of clinical improvement were seen in various parameters: a 13.3% elevation in oxygen saturation elevation, a 35.1% decline in RR, a 31.5% decrease in pulse rate, and an 18.6% and 17.6% decrease in sBP and dBP, respectively. Moreover, after the values of several clinical signs (RR, heart rate, O2%, sBP and dBP) underwent a significant improvement (mostly for a period of 32–40 hours) during treatment, they did not manifest any further detectable clinical signs and did not change significantly during further monitoring. For example, after oxygen saturation had reached 93% (normal) following 32–40 hours of monitoring and treatment, it did not change significantly during further follow-up until the 56^th^ hour (the 8^th^ measurement). In contrast, the post-treatment ITI values of the study group were lower in comparison to the non-significant ITI fluctuations in the control group, indicating significant residual fluid volume, which, however did not cause clinical symptoms, especially at rest position. The small ITI changes in the control group may be explained by stress-inducing factors of the active disease (e.g., fever, dyspnea, etc.).

The similarity of the relatively narrow values of the initial ITI in each of the two groups (~33 ohm in study group and ~59 ohm in the control group) indicates that the baseline condition of the right lung was similar among all the patients in each group. The ITI levels in patients with pleural effusion were much lower than were the fluctuations of ITI during the monitoring of the controls. The average baseline differences of the ITI between the two groups was ~26 ohm, and it decreased to ~14 ohm after treatment. This is in comparison to pulmonary edema at the peak, where the mean difference between patients and controls was 21 ohm [8]. The reliability of ITI measurement as a tool for detecting and monitoring pleural effusion was supported by a high correlation of ITI values measured at the outset with all parameters used to evaluate the patients’ clinical condition. These correlations remained significant at least after 32 hours of monitoring in the study group.

As mentioned earlier, ITI changes can detect pleural effusion in the absence of overt clinical symptoms, as is the case for pulmonary edema [15]. ITI measurements can diagnose subclinical pulmonary edema about 1 hour before overt edema. In comparison, because pleural effusion develops relatively slowly, we consider that ITI methodology is capable of identifying the presence of pleural fluid much earlier than 1 hour before the appearance of clinical symptoms and signs. This finding is very important for early diagnosis of non-malignant pleural effusion and early initiation of treatment, especially in patients with heart failure, the most common cardiac syndrome and one that can be effectively treated by diuretics in an ambulatory setting. Early detection and treatment can prevent life-threatening respiratory distress. We think that the ITI of ambulatory patients can be measured once daily by the RS-207 monitor much in the same way that BP is measured, and that the patient can seek and receive appropriate treatment in the event that there is a decrease in impedance below acceptable values.

## Conclusions

The measurement of ITI is a highly sensitive noninvasive methodology and one that may be especially useful for ambulatory patients with recurrent pleural effusion of different etiologies. It may also enable early diagnosis and early implementation of treatment of yet asymptomatic or not life-threatening dyspnea, prevent respiratory distress and obviate the need for mechanical ventilation. ITI monitoring with the intent to treat upon an ITI result of 33 ohm or less may enable the initiation of early effective therapy. We contend that the EGM monitor can be used in the future for both hospitalized patients and outpatients with moderate or severe pleural effusion from different etiologies, and enable the latter to receive preventive treatment with no need for hospitalization.

## Supporting Information

S1 CONSORT Checklist (DOC)

S1 File Clinical Trial Registration. (MHT)

S2 File Local Ethical Committee. (PDF)

S1 Protocol Study Protocol. (DOC)
