# Supplementary material for: Internal Thoracic Impedance - A Useful Method for Expedient Detection and Convenient Monitoring of Pleural Effusion
Source: PLoS One. 2015 Apr 28;10(4):e0122576. doi: 10.1371/journal.pone.0122576 (PMC4412530; doi:10.1371/journal.pone.0122576)
Supplement: S2 File — (PDF) [file pone.0122576.s003.pdf]

מדינת ישראל
STATE OF ISRAEL

Ministry of Health
Pharmaceutical Administration
Clinical Trials Department
Medical Devices & Advanced Therapies

משרד הבריאות
אגף הרוקחות
המחלקה לניסויים קלינים
אמ״ר, תאים ורקמות וטיפול גני

טופס 8
אישור מנהל משרד הבריאות
לפי תקנות בריאות-העם (נסויים רפואיים בבני-אדם) התשמ"א 1980

תאריך : 26.3.2012

לכבוד
פרופ' מרסל טופילסקי
יו"ר ועדת הלסינקי
בית חולים איכילוב
תל-אביב, 64239

נכבדי,

הנדון: **אישור לביצוע ניסוי רפואי בבני אדם**
סמוכין: אישור ועדת הלסינקי מיום: 28/10/2011

מס' האישור: **HTA6166**
מס' הבקשה: **HT6166**
שם החוקר הראשי: דר' חר"ך גדעון, פנימית ג' .
שם האמר: **Edema Guard Monitor (Model RS-207).**
שם היצרן: **R.S. Medical Monitoring.**
שם הניסוי הרפואי: DETECTION OF PLEURAL EFFUSION BY INTERNAL THORACIC IMPEDANCE METHOD. בקשה מוסדית מס' 0504-11

מסמכי הניסוי: פרוטוקול סימון גרסה 2 מתאריך 7.11.2011; טופס הסכמה גרסה 4 מה-21.11.2011; נספח 1 מתאריך 2.10.2011; נספח לטופס הסכמה (ציור הדרכה להדבקת האלקטרודות) מתאריך גרסה 2 מה-7.11.2011; תקציר פרוטוקול מתאריך 7.11.2011 (גרסה 2);

החלטה: הבקשה לביצוע הניסוי הרפואי אושרה בתוקף סמכותי כמנהל.

הערה כללית: במקרה של התקשרות מסחרית לביצוע הניסוי הרפואי בין הי וזם, החוקר הראשי והמוסד הרפואי, רשאי מנהל המוסד הרפואי להנפיק אישור לניסוי לאחר שקבע כי חוזה ההתקשרות עומד בכל דרישות הנוהל לניסויים רפואיים בבני אדם של משרד הבריאות (1999).

בכבוד רב,

ד"ר קתרין אלה
רכזת ארצית לניסויים קליניים באמ"ר
תאים ורקמות וטיפול גני

העתק:
דר' אסנת לוקסנבורג, ראש מינהל טכנולוגיות רפואיות ותשתיות
מגר' בתיה הרן, מנהלת אגף הרוקחות
דר' נדב שפר, מנהל מחלקת האמ"ר

29 Rivka Str.
P.O.B. 1176
Jerusalem 91010

Tel. 972-2-5681216/356 :טל
FAX 972-2-6725827 :פקס
www.health.gov.il/drugs
clinical.trials.devices@moh.health.gov.il

רח' רבקה 29 קומה 2
ת.ד. 1176
ירושלים 91010
