# Supplementary material for: Internal Thoracic Impedance - A Useful Method for Expedient Detection and Convenient Monitoring of Pleural Effusion
Source: PLoS One. 2015 Apr 28;10(4):e0122576. doi: 10.1371/journal.pone.0122576 (PMC4412530; doi:10.1371/journal.pone.0122576)
Supplement: S1 Protocol — (DOC) [file pone.0122576.s004.doc]

**Protocol of the study . 07.11.2011**

**Detection of pleural effusion by internal thoracic impedance method**

Gideon Charach, MD , Olga Metiuch.

**Introduction**

Early detection of pleural effusion (PLE) would improve the treatment. However, preclinical detection of pleural effusion is often not possible.

Radiographic examination, widely used for detecting pleural effusion ,is not suitable for prolonged monitoring of patients at high risk of developing PLE especially ambulatory or at home (1-9).

The currently available methods for monitoring and early detection such as the measurement of pulmonary capillary wedge pressure or measurement by double

indicator thermodilution, are not reliable enough and may themselves lead to complications(2-8).

Yu C-M et al.(9) recently reported successful prediction of CPE by a surgically implanted impedance plethysmograph integrated into a pacemaker . The system was shown to be sufficiently sensitive for cardiogenic pulmonary edema preceding. However this method is invasive and not suitable for wide use (9).

Therefore, these methods are seldom employed for the detection of PLE (3–7).

Thus, a safe and accurate technique for early detection and monitoring of PlEf would be of great clinical value. The changes in the blood and extravascular fluid content in the lungs in humans and animals can be easily monitored using a noninvasive and totally safe procedure based on impedance plethysmography (5-12-). This method is based on the fact that the impedance of the lungs decreases with the increase in the fluid and free ions content in them (8,13,). However, it proved to be unsuitable
for preclinical stage monitoring of PLE (2-8) for several reasons, mainly the low sensitivity of the existing impedance plethysmographs. This is due to the high skin-electrode contact resistance (13,14), which is of an order of magnitude high erthan the impedance of the lung.

After cleaning the skin of fats with alcohol and moistening it with electrode paste, the value of the skin electrode contact resistance is approximately 400–

500 ohm (11-13).

Therefore, for two measuring electrodes (front and back of the chest), this value must be 800–1000 ohm.

Total transthoracic impedance (TTI) consists of internal thoracic impedance (ITI) and skin contact impedance. The TTI may vary from 920 to 1230 ohm in different individuals (11-13). However, change of TTI in PLE was reported by others to vary in the range of 2–16 ohm (7-9, 11-18), which is approximately1% of TTI and 1.5% of skin contact impedance. Monitors used in previous works were not sensitive enough to detect the relatively small changes in pulmonary impedance(2-7).
In addition, during prolonged monitoring, the skin-electrode contact impedance may also change (13,14) due to penetration of the ions from sweat into the electrode paste and drying of the paste. It may significantly exceed the change in the impedance of the lung caused by the development of cardiogenic pulmonary edema (2-7). Kubicek et al. (10) attempted to develop a method in which a tetrapolar electrode system is used to overcome this problem.

However, because this system measures the impedance of the entire chest, a large part of the electrical field is concentrated in the surface tissues, and this considerably reduces the sensitivity of the method (10,11). Furthermore, Kubicek’s electrodes may be burdensome and for critically ill patients when prolonged monitoring is required. The main disadvantage of this and other more sensitive methods is surgical implantation of the electrodes (10,13)
A new impedance monitor, model RS- 207 EDEMA GUARD MONITOR (R. S. Medical Monitoring, Jerusalem, Israel), has been developed (15). The monitor design has also solved the problem of the drift skin-to-electrode electrical resistance and its drift during prolonged monitoring by separating ITI from skin-to-electrode electrical resistance. The result of the above mentioned actions is a value of Internal Thoracic Electrical Resistance (Impedance)- ITI. The method of ITI estimation that completely described by Rabinovich (15) Unlike the existing impedance monitors,

the new monitor measures only ITI, which roughly equals lung impedance, by automatically calculating skin electrode impedance and subtracting it from TTI. According to the published data, ITI ranges from 40 to 100 ohm in individuals without CPE and, on average, decreases to 30 ohm, i.e., by30% to 75% during CPE (11-12,16-21). These results showed much higher sensitivity compared with the 1.5% changes found using TTI methods. This is an average 35- fold increase (range, 20- to 50-fold intraindividual constitutional differences) of impedance plethysmograph sensitivity compared with the method without substraction of skin electrode resistance (2-8). This monitor was succefully used for early detection of pulmonary edema however there was no any expearence on pleural fluid detection.(11,12,16-20.)

The aim of the present study is to evaluate the suitability of the RS-207 in monitoring PlEf at clinical and preclinical stage. The tests will be considered successful if ITI will be low or decreased parallel to or before the appearance of clinical signs and radiograph findings compatible with PlEf, and then demonstrably increased during

their resolution.

**Considerations and purpose**

Previous study based on measurements of ITI in early diagnosis of pulmonary edema showed suitability of the RS-207.

There is no any study that deals with early diagnosis of pleural effusion by this simple nonexpensive method which can be used in ICU and ambulatory setting.

Measuring ITI, the main component of which is lung impedance, is a noninvasive and safe method. PLE will be diagnosed in accordance with well-accepted clinical signs (dyspnea, cyanosis, pulmonary rales, crepitations, arterial hypoxemia) and roentgenological criteria.

The consideration of the study : evaluation of the suitability of the RS-205 monitor in detecting the PLE at preclinical stage before the overt appearance of clinical signs for assessment of early treatment and then to demonstrate increased of ITI during their resolution.

**MATERIALS AND METHODS**

Study design:

Prospective, controlled study, 100 patients

Patients.

Fifty consecutive patients admitted to our Department of Internal Medicine C with PLE will be recruited to the study according to the inclusion and exclusion criteria detailed below.Fifty carefully matched patients without CPE will be select for the control group.All participants will give written informed consent before entering the study.

Inclusion criteria for study group **will be** as follows: patients 40-90 year old **with pleural effusion on chest X-ray** of different etiologies: coronary heart disease-(CHD), valvular heart disease, renal failure, infectious disease malignant diseases( all complicated by pleural effusion).

The control group **will be** comprised of patients 40-90years old without pleural effusion established by chest X-ray.

The exclusion criteria **will be** respiratory failure due to diseases, wearing a **pacemaker**, thoracic deformation, pulmonary edema and embolism.

The protocol of the investigation corresponded to the principles outlined in the Declaration of Helsinki, and the study protocol will be approved by the local Ethical Commission of the participating medical centers and the Israeli Ministry of Health.

Method of ITI measurement

ITI will be measured by the RS-207 impedance monitor (RSMM LTD, Jerusalem, Israel). Standard 1-cm electrocardiographic (ECG) electrodes (Nice Medical Products, Type 4500 foam, TUV, Rheinland) will be attached to the skin after it had been shaved and cleansed with alcohol. Three electrodes will be placed in a straight vertical line on the anterior surface of the right chest, along the edge of the sternum (Fig. 1). The central measuring electrode (#2) will be placed at the second intercostal space. Three other electrodes will be placed in a straight horizontal line on the back of the right chest along the 8^th^ intercostal space (Fig. 1). The central electrode (#5) will be placed at the point where the 8^th^ intercostal space crossed the scapular line. The reference electrodes #1, 3, 4 and 6 will be attached to the chest in pairs in line with and on each side of electrodes #2 and 5. All six electrodes will be connected to the RS-205 monitor.

The diagnosis of PLE will be based on the following symptoms and signs: progressive dyspnea at rest (20 respirations), tachycardia (90 beats/min), diaphoresis, cyanosis, dullness on percussion, crepitation rales, a roentgenogram pleural effusion.

and arterial hypoxemia (92%) (15). The definitive diagnosis of PLE in this study will be based on roentgenogram findings(galld standard).

**ITI measurments will be monitored continuosly. Clinical examination, and measurements of** oxygen saturation ( O_2_%),respiratory rate, pulse, systolic (sBP) and diastolic blood pressure (dBP) evaluation were to be repeated every 8 hours until monitoring were terminated when successful conservative treatment or pleural **puncture will be achieved. Follow-up chest x-ray was performed thereafter**

. Additional roentgenograms will be performed whenever the patient’s condition changed. Clinical examinations, roentgenogram,and will be performed when monitoring will be discontinued.

Patients with PLE will be treated conventionally **(not according the monitor findings)** 1.conservatively mainly based on the diuretics: furasemid and aldospirone or 2.pleral punction.

Monitoring of pleural effusion is carried out by placing three electrodes on the

front and three electrodes on the back of the thorax (Fig. 1)

The **Edema Guard Monitor (EGM)** model RS-207 (RS Medical Monitoring Ltd, Jerusalem, Israel) [ 14 ] measure unlike the technique used in the existed noninvasive plethysmographs the electrical resistance **across of right half of thorax not along of the thorax**. Thus the EGM's measured electrical field is moved away from the large arteries to the area of the right lung (Figs. 1 and 2). The values of skin-measuring electrode impedances received on the front and on the back of the thorax are subtracted from trans thoracic impedance(TTI) and thus we arrive at the value ITI (Fig. 1) by special algorithm (11,14,15).

Fig. 1

| 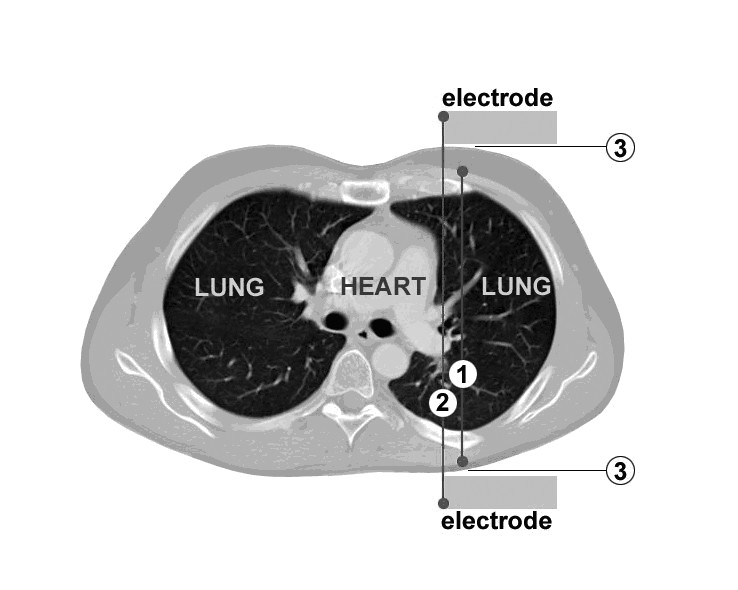 | **Cross‑section of the thorax**  **1** – **Internal thoracic impedance, 50 - 80 Ohm**  **2** –**Transthoracic impedance, 1050 – 1280 Ohm**  **3** – **Skin-electrode impedance, 500 – 600 Ohm** |
| --- | --- |

Fig. 2


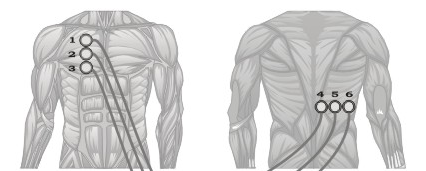


**Investigator s part**: to measure the ITI by RS 207 monitor on admission and during the hospitalization and before discharge. All values will be included in report chart. Student will take relevant anamnestic data, respiration rate and pulse oxymetry(oxygen saturation)- on the same occasions when the ITI measurements will be taken.

Clinical signs of pleural effusion will be examined by supervisor (tutor) together with the student.

**Importance of the work**: Early detection of pleural effusion would improve the treatment. However, preclinical detection of pleural effusion is often not possible.

The internal thoracic impedance measurement is high sensitive non invasive and useful method for detection and monitoring of treatment of patients with

PLE and allows earlier and more effective therapy (**by diuretics or pleural punction**) and can prevent respiratory distress or mechanical ventilation . **This hypothesis based on our previous studies in patients with heart failure and pulmonary edema who received the treatment on preclinical stage of pulmonary congestion and didn't development pulmonary edema. (11,17-21).**

The method may be especially useful for ambulatory ( at home) patients with recurrent pleural effusion of different etiologies.

**REFERENCES**

1. Luepker RV, Michael JR, Warbasse JR. Transthoracic electrical impedance:
    Quantitative evaluation of non-invasive measure of thoracic fluid volume. Am
    Heart J 1973; 85:83-93.
2. Fein A, Grossman RF, Jones G, Goodman PC, Murray JF. Evaluation of
    transthoracic electrical impedance in the diagnosis of pulmonary edema.
    Circulation 1979; 60:1156-1160.
3. Saunders CE. The use of transthoracic electrical bioimpedance in assessing
    thoracic fluid status in emergency department patients. Am J Emerg Med 1988;
    6:337-340.
4. Spinale FG, Reines HD, Cook MC, Crawford FA. Noninvasive estimation of
    extravascular lung water. J Surg Res 1989; 47:535-540.
5. Zellner JL, Spinale FG, Crawford FA. Bioimpedance: A novel method for the
    determination of extravascular lung water. J Surg Res 1990; 48:454-459.
6. Campbell JH, Harris ND, Zhang F, Morice AH, Brown BH. Detection of changes
    in intrathoracic fluid in man using electrical impedance tomography. Clin Sci

(Lond) 1994; 87:97-101.

1. Newell JC, Edis PM, Ren X, Larson-Wiseman JL, Danyleiko MD. Assessment of
    acute pulmonary edema in dogs by electrical impedance imaging. IEEE Trans
    Biomed Eng 1996; 43:133-138.
2. Nierman DM, Eisen DI, Fein ED, Hannon E, Mechanick JI, Benjamin E.
    Transthoracic bioimpedance can measure extra vascular lung water in acute lung
    injury. J Surg Res 1996; 65:101-108.
3. Yu C-M, Wang L, Chau E, et al: Intrathoracic impedance monitoring in patients with heart failure. Circulation 2005;112 841-8.
4. Kubicek WG, Patterson RP, Witsoe DA. Impedance cardiography as a
    noninvasive method of monitoring cardiac function and other parameters of the
    cardiovascular system. Ann NY Acad Sci. International conference of

bioelectrical impedance. 1970, 170, Art. 2, p. 724 – 731.

1. Charach G, Rabinovich P, Grosskopf I, Weintraub M: Transthoracic monitoring
    of the impedance of the right lung in patients with cardiogenic pulmonary edema.
    *Crit Care Med* 2001;29 1137-44.

12. Shochat M, Meisel S, Rabinovich P, Peled B. Monitoring of the internal
 thoracic impedance: a novel method to detect pulmonary edema before
 appearance of clinical signs. *Supp. to J of the American College of
 Cardiology.* *52^nd^ Annual Scientific Session*. 2003; 1206-73

13. Yamamoto Y, Yamamoto T: Characteristics of skin admittance for dry
 electrodes and measurement of skin moisture. Med Biol Eng Comput
 1986;24 71-7.

**14**. Yamamoto T, Yamamoto Y: Electrical properties of the epidermal
 stratum corneum. *Med Biol Eng* 1976;14 151-8.

15. Rabinovich P, Shochat M, Zeldin V. Milman O. Method and device for
 stable impedance plethysmography. US Patent No. 5,749,369; May 12,
 1998.

16. Shochat M, Meisel S, Rabinovich P, Peled B, Shotan A: A new method
 for detecting cardiogenic pulmonary edema before appearance of clinical
 signs and for the evaluation of treatment efficacy. *Supp. to J of the
 American College of Cardiology. 53^nd^ Annual Scientific Session:* 2004;
 1154-96

17. Shochat M, Charach G, Frimerman A, Rabinovich P, Shotan A, Meisel S:
 Internal thoracic impedance monitoring: a new prospect in acute heart
 failure. *Eur Heart J* 2004; 25: Suppl. Abstract 72: 500.

*1*8. Shochat M, Kazatzker M, Charach G, et al: Internal thoracic impedance
 monitoring: a new tool for the early diagnosis and treatment of acute
 heart failure. *Eur J Heart Fail 2005; 4: Suppl 1*, Abstract 354:79-80.

19. Shochat M, Charach G, Meyler S, et al: Internal thoracic impedance monitoring: a novel method for the early detection of cardiogenic pulmonary congestion at the pre-clinical stage. *Cardiovasc Revasc Med* 2006;7 41-5.

# 20. Shochat M, Charach G, Meyler S, et al: Prediction of cardiogenic pulmonary

# edema onset by monitoring right lung impedance. Intensive Care Med 2006; 32

# 1214-21.

# 21 [**Shochat M**](http://www.ncbi.nlm.nih.gov/pubmed?term="Shochat M"%5BAuthor%5D), [**Shotan A**](http://www.ncbi.nlm.nih.gov/pubmed?term="Shotan A"%5BAuthor%5D), [**Trachtengerts V**](http://www.ncbi.nlm.nih.gov/pubmed?term="Trachtengerts V"%5BAuthor%5D), [**Blondheim DS**](http://www.ncbi.nlm.nih.gov/pubmed?term="Blondheim DS"%5BAuthor%5D), [**Kazatsker M**](http://www.ncbi.nlm.nih.gov/pubmed?term="Kazatsker M"%5BAuthor%5D), [**Gurovich**](http://www.ncbi.nlm.nih.gov/pubmed?term="Gurovich V"%5BAuthor%5D)

# [**V**](http://www.ncbi.nlm.nih.gov/pubmed?term="Gurovich V"%5BAuthor%5D), [**Asif A**](http://www.ncbi.nlm.nih.gov/pubmed?term="Asif A"%5BAuthor%5D), [**Shochat I**](http://www.ncbi.nlm.nih.gov/pubmed?term="Shochat I"%5BAuthor%5D), [**Rozenman Y**](http://www.ncbi.nlm.nih.gov/pubmed?term="Rozenman Y"%5BAuthor%5D), [**Meisel SR**](http://www.ncbi.nlm.nih.gov/pubmed?term="Meisel SR"%5BAuthor%5D)

# A novel radiological score to assess lung fluid content during evolving acute

# heart failure in the course of acute myocardial infarction.

AC Card Care 2011; 13(2): 81-86
